# Precision nutrition-based strategy for management of human diseases and healthy aging: current progress and challenges forward

**DOI:** 10.3389/fnut.2024.1427608

**Published:** 2024-08-09

**Authors:** Vipin Kumar Singh, Xiao-Hu Hu, Amit Kishore Singh, Manoj Kumar Solanki, Pooja Vijayaraghavan, Rajpal Srivastav, Naveen Kumar Joshi, Madhuree Kumari, Sandeep Kumar Singh, Zhen Wang, Ajay Kumar

**Affiliations:** ^1^Department of Botany, K.S. Saket P.G. College, Ayodhya, India; ^2^Guangxi Key Laboratory of Agricultural Resources Chemistry and Biotechnology, Agricultural College, Yulin Normal University, Yulin, China; ^3^Botany Department, B.N. College, T.M. Bhagalpur University, Bhagalpur, India; ^4^Department of Life Sciences and Biological Sciences, IES University, Bhopal, Madhya Pradesh, India; ^5^Amity Institute of Biotechnology, Amity University, Noida, India; ^6^Amity Institute of Microbial Biotechnology, Amity University, Noida, India; ^7^Department of Biochemistry, Indian Institute of Science, Bengaluru, India; ^8^Department of Microbiology, Indian Agriculture Research Institute, New Delhi, India

**Keywords:** biomarker, microbiota, bioactive constituents, machine learning, algorithm, immunity

## Abstract

Currently, the treatment of various human ailments is based on different therapeutic approaches including traditional and modern medicine systems. Precision nutrition has come into existence as an emerging approach considering the diverse aspects such as age, sex, genetic and epigenetic makeup, apart from the pathophysiological conditions. The continuously and gradually evolving disciplines of genomics about nutrition have elucidated the importance of genetic variations, epigenetic information, and expression of myriads of genes in disease progression apart from the involvement in modulating therapeutic responses. Further, the investigations have presented the considerable role of gut microbiota comprising of commensal and symbionts performing innumerable activities such as release of bioactive molecules, defense against pathogenic microbes, and regulation of immunity. Noteworthy, the characteristics of the microbiome change depending on host attributes, environmental factors, and habitat, in addition to diet, and therefore can be employed as a biomarker to unravel the response to given food. The specific diet and the components thereof can be suggested for supporting the enrichment of the desired microbial community to some extent as an important part of precision nutrition to achieve not only the goal of human health but also of healthy aging.

## 1 Introduction

The rising pressure of different disorders including chronic diseases ensuing from ever changing complex epidemiological events has long been highlighted (1) to pose risks to health and life span (2) in addition to those resulting from mineral imbalances. Surprisingly, the mortality and disabilities caused by chronic diseases are comparatively higher than infectious diseases and injuries (3). The risk factors for such diseases are identified as lifestyle modification at the individual level, variation in diet at global scale, declining physical activities, lack of preventive measures, and nutrient deficient foods. Noteworthy, sufficient intake of nutrients promotes good health, whereas, the deficiency of essential nutrients has tens diverse diseases (4). Therefore, several developed countries like United States, European Union, across the globe have made the implementation of essential minerals and vitamins mandatory. Apart from the suggestion of recommended diets comprising of plant and animal based products such as vegetables and meat, providing essential minerals, amino acids, vitamins, and fatty acids known to be required for sustaining the health of a given populace. Moreover, the continued qualitative and quantitative modifications in human diet owing to technological advances meant for food generation as well as processing have resulted in substantial perturbations of metabolic activities leading to varied human health disorders (5–7) including cancer, allergies, and cardiovascular diseases. Such perturbations resulting from modulations in dietary contents are much pronounced in developed countries.

The alleviation and treatment of human diseases can be divided broadly into traditional (Ayurveda, Unani, Siddha, etc.) and modern medicine systems (Allopathic). Traditional medicine relying to a greater extent on natural products of plant origin has long been recognized for treating different human disorders (8, 9). The development of strategies for prevention of innumerable diseases through improvement of immunity as well as offering the ways for produce valuable drugs (10, 11) is important approach of disease management. Traditional medicine has also been differently referred to as complementary and alternative medicine (12). The modern medicine is characteristically marked by the continued advances in technology aimed toward refining diagnostic methodologies and treatment of disorders started from 1946 to the current period (13). Modern medicine employing a single or mixture of a few purified bioactive compounds could have profound undesirable effects in comparison to traditional medicine generally prescribing crude mixtures of plants causing minimum side effects rendered by modulatory action of other components. Further, the isolated bioactive used in modern medicine systems to treat human health disorders should rather be used for deciphering the mechanism of action (14).

Precision nutrition is an emerging research frontier for managing health and treating human diseases through extensive investigations on bodily metabolic responses so as to suggest optimum dietary plans (15). The science of precision nutrition involves major factors such as environmental exposures, lifestyle, plausible interaction between gene and nutritional components (nutrigenomics), gut microbial communities, and processes influencing gene expression not only at the intra-individual level, but also at the inter-individual level (16, 17). The implementation of precision nutrition can be successful through analysis of individual features, continuous nutrient availability, and control as well as site-specific delivery (16), in addition to the introduction of different omics technologies.

**Figure 1:**
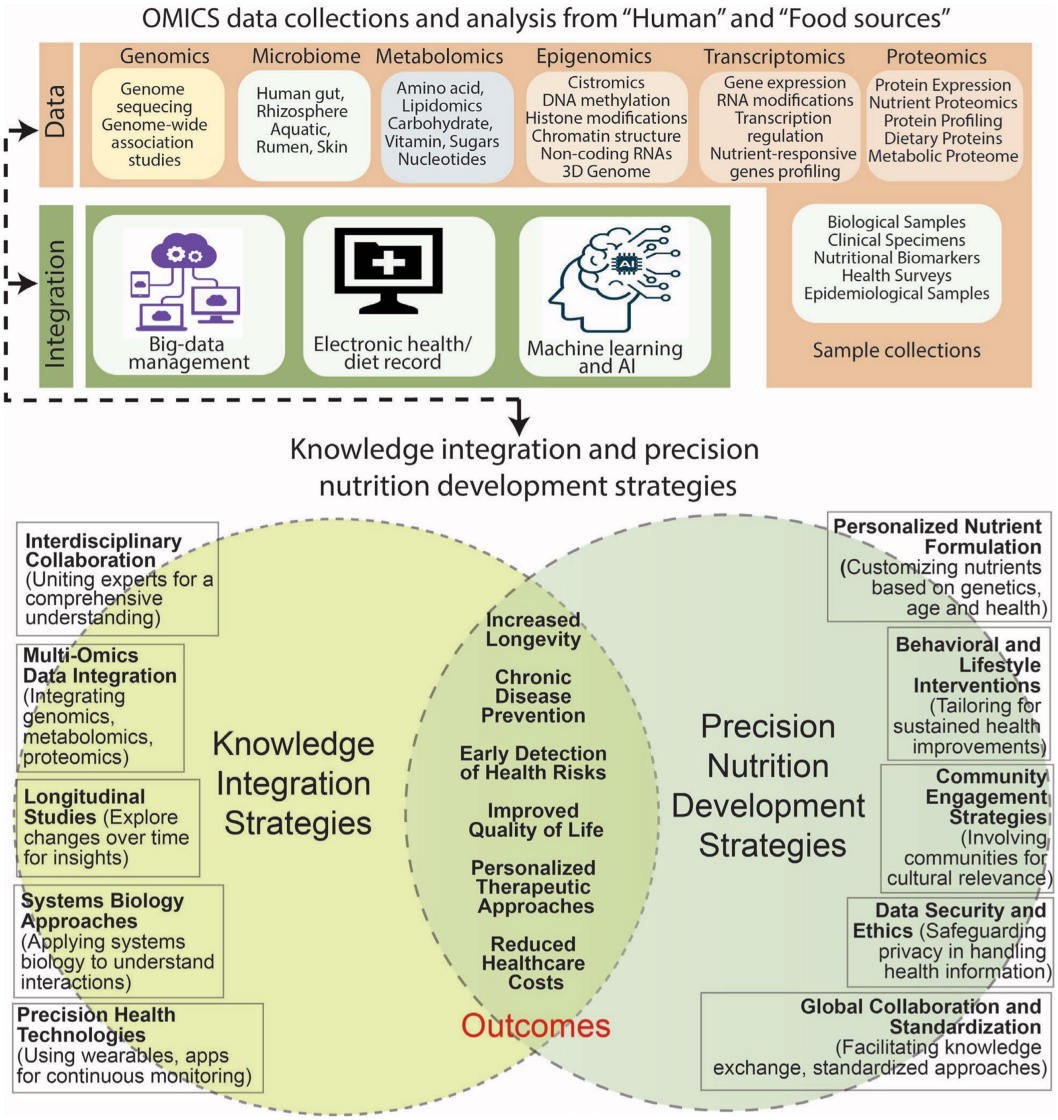
Strategies in knowledge integration and precision nutrition against aging and diseases involve interdisciplinary collaboration, multi-omics data integration, longitudinal studies, systems biology, and precision health technologies. The development includes personalized nutrient formulation, behavioral interventions, community engagement, data security, and global collaboration. Major outcomes: increased longevity, disease prevention, improved quality of life, reduced healthcare costs, and personalized therapeutic approaches.

Recently, the contribution of metabolomics in achieving the targets of precision nutrition has been reviewed by Brennan and de Roos (17). In this context, the strategy of metabolomics may comprise analyzing metabolic constituents (18) to reveal the knowledge concerning the effect of nutrients on metabolic activities, and available bioactive molecules. The variations in metabolic profiles, therefore, can be used for identifying metabotypes to advise diet plan. Furthermore, the data available on metabolite patterns can be combined with additional factors to gain insight into an individual’s response to dietary intake. The precise identification and analysis of metabolites using mass spectrometry (MS), infra-red (IR), and Raman spectroscopy, as well as nuclear magnetic resonance (NMR) in combination with modern computational approaches, can further advance the progress in the discipline of metabolomics (19, 20). Additionally, different approaches including nutrigenetics, epigenetics, and nutriepigenetics (21, 22), proteomics (23), gut microbiota (24), dietary interventions (25), nutraceuticals (26), lifestyle (27), and machine learning tools (28) have shown promise in the field of precision nutrition focused toward better human health (Figure 1).

Most of the previous articles have included only a few aspects of precision nutrition. The holistic information to our knowledge is still lacking. Therefore, the present review has attempted to provide updated information about different strategies adopted for precision nutrition. The limitations and future opportunities in the discipline of precision nutrition given good human health are finally discussed.

## 2 Different omics technology and precision nutrition

### 2.1 Nutrigenomics and management of human health in the era of the 21st century

The successful launch of human genomics in the 2000s led to the emergence of novel research frontier including nutrigenomics which deals with the study of interaction between dietary components and the genome, proteome, and metabolome. However, the continuous development of several omics like metabolomics, proteomics genomics, etc. has provided ample information not only for nutrigenomics but also mainstream to the nutritional values at individual level recognized as precision nutrition.

Nutrients are environmental factors having (i) the potential to alter gene expression, and (ii) increase the risk of diseases through impairing the epigenome of the developing organisms. Exceedingly increasing interest in such interactions led to the emergence of nutrition genetics or nutrigenetics, which allow us to understand the relation between diet and gene expression although they follow different approaches. Nutrigenomics deals with the study of the effect of nutrients on gene expression, while nutrigenetics describes the cellular response to the nutritional components apart from other external factors by observing metabolism and the site of action (29).

### 2.2 Metabolomic profiles (metabolizing)

Different metabolic constituents can be regarded as biomarkers to provide real-time information about the dietary food intake and quality of the food consumed by the individual (17). Clark et al. (30) in a review reported the potential biomarkers for whole grains, soy, and sugar that have been validated primarily based on several criteria such as biological plausibility, time and dose–response, robustness, reliability, stability, and performance of the method which have used to explore the biomarker potential.

Recently, some of the studies have indicated the use of biomarkers as a tool for establishing dietary patterns. For example, multiple plasma metabolites have been used as a signature biomarker in the Mediterranean diet and further assessed for their association with cardiovascular disease (CVD) risk (31). Furthermore, several studies also indicated the influence of metabolic profile-based biomarkers in the assessment of disease risk (32). However, it has been concluded that factors such as age, body mass index (BMI), etc., play a significant role in influencing the potential of the biomarker of the individual.

The metabolomics approach is further applied to classify the individuals based on their metabolic profiles. This approach allows for the comprehensive analysis of metabolites, thereby providing a detailed understanding of the metabolic processes and pathways active in different. In precision nutrition, metabotyping plays a vital role in the development of tailored precision nutrition via categorizing individuals based on their unique metabolic profiles (33). Generally, the metabolizing approach is based on several biomarkers (triacylglycerol, cholesterol, HDL-cholesterol, and glucose) to classify the population of definite size into metabotypes that are further subjected to delivering tailored advice (34). However, the concept of metabotypes further needs to be redefined considering the dietary requirements and the economic status of the population because these factors significantly influence metabolic profiles and health outcomes.

### 2.3 Metagenomic profiles

Development of cutting-edge sequencing tools like next-generation sequencing (NGS)which explore the hidden information concerning spatial and temporal variation of microbiota present in any biological samples. Metagenomic profiling of the gut microbiome of an individual’s mid gut play a vital role in nutrigenomics. Furthermore, studies indicated that gut microbiota changes from birth to successive stages (35). Besides this, microbiome profiling also helps to decipher the type of food preferred by certain age groups. The presence of Firmicutes- and Bacteroides-dominant patterns in adult age groups indicated the long-term ingestion of carbohydrate and animal fat and/or protein-rich diet, respectively (35, 36). These findings revealed how factors like age, and food habits affect the structure and functions of gut microbiome dynamics and therefore should be considered while handling metagenomic data. However, integration of other “omics” such as genomics, transcriptomics and metabolomics will provide more comprehensive overview of an individual variability in response to diet and enables the development of personalized nutritional strategies that are more effective in promoting health and preventing disease.

### 2.4 Nutrigenetics

The interaction of personalized diet or nutrition and genes with the objective of achieving sound health is the ultimate goal of nutrigenetics. Nutrients that we intake are one of the environmental factors that interact with the genes and affect the DNA metabolism, repair mechanism, and gene expression of individuals. Recent published reports further confirmed that single nucleotide polymorphisms (SNPs) can be used as molecular tools to gain the information on the interaction between human diseases and the nutrition diet (37). For example, phenylketonuria (PKU) disorder is the result of altered gene expression by SNPs, interaction with metabolite leads to the deficiency of the phenylalanine hydroxylase gene (38). Other examples of polymorphism include Lactase-phlorizin hydrolase gene (LPH) polymorphism, glutathione peroxide gene polymorphism, Manganese superoxide dismutase (MnSOD) in which substitution or deletion of the specific genes resulted in an increased risk of liver and breast cancer, respectively (39). There is growing evidence that multiple pathways may be involved in the cause of multiple diseases where transcription factors alter gene expression patterns via interaction with bioactive food components (40). The influence of transcriptomic on gene expression is not only dose-dependent but also time-dependent. Future aspects of nutrigenetics can be helpful in knowing which genetic factors should be given to particular genetic subgroups that may reduce the risk of emergence of chronic diseases.

### 2.5 Nutriepigenetics

Epigenetics involves the switch on or switch off the gene expression without affecting the DNA sequences. In general, epigenetics involves DNA methylation, histone modifications, and non-codingRNA regulation to influence cellular metabolism. Bioactive food components such as micronutrients and chemicals are known to be involved in DNA methylation (41). Several studies indicated the role of dietary factors in the provision of methyl for the formation of S-adenosylmethionine that may alter the DNA methyltransferase activity and impair folate metabolism. Disruption in foliate metabolism eventually causes risk of cardiovascular diseases, neurological disorders, and other developmental anomalies (41, 42). In addition to DNA methylation, the role of non-coding RNAs [microRNAs (miRNAs)] and long noncoding RNAs (lncRNAs), has also been reported in the epigenetic process. Recently, miRNAs and lncRNAs, are known to have links with chronic disorders like cardiovascular diseases and obesity (43). In nutshell, DNA methylation and miRNAs can be treated as target markers for future precision dietary interventions.

## 3 The role of microorganisms (gut microbiome)

### 3.1 Human microbiome and role in human health management

The human microbiome influences human physiology through different mechanisms. The gut microbiome regulates digestion, metabolism, colonization resistance, immune modulation, and cellular functions in various ways (44). The dysregulation of microbial community functions may induce inflammations and disease development. The pathogenesis of opportunistic microbes may occur in these conditions of gut dysbiosis. However, imbalance in the composition of normal gut microbiota represents dysfunction or disorders (45). The association of gut microbiota with human health and diseases is now evident by various investigations (46). The existence of gut-brain and gut-lung axis is now correlated with illnesses, and associated with brain and respiratory tracts respectively, suggesting the importance of gut microbes in human health. However, there are still many aspects to explore and to better understand the association between gut microbiome and human health in complex disorders including cancers and other serious illnesses like respiratory illness, i.e., COVID-19 infections.

Current disease-specific therapeutic approaches may miss the large portion of the microbial populations, whose impact may be significant on human health. Thus, personalized medicine has been focused on therapeutics now (45). The personalized medicine is a future path of diagnostics and therapeutics as it takes care of individuals’ physiological and genetic factors (45, 47). Further, each patient has different biological habits, lifestyle, metabolic profile, food environments, and gut microbiome. So, precision nutrition could help design comprehensive diets plans based on individual variables. It also takes care of socioeconomic parameters during recommendations of diet plans (48). Further, the modern lifestyle has influenced our dietary patterns and food habits adversely leading to obesity and other related health problems (49). Therefore, the dietary recommendations can play an important role in disease management. The role of personalized nutrition is to manage diseases by diet monitoring and recommendations (50). There are many layers of the dietary recommendations. The genetic and biological characteristics of the individual are taken care of while determining the dietary recommendations in personalized nutrition (45). The fat content of the diet, and other modifications in obese or diabetic individuals is one of the such interventions while fixing for the course of personalized medicine. The same type of the diet may have different metabolic responses in different patients. The difference in glycemic responses in different patients has also been observed (51). The gut microbiome affects human metabolism in different ways and thus influences the manifestation of the diseases.

The chronic disease of arteries identified as atherosclerosis is responsible for significant mortality worldwide. The use of hypoglycemic, antiplatelet, and lipid lowering drugs are the current therapies for the treatment of atherosclerosis (52). The role of trimethylamine-N-oxide (TMAO) is associated with gut microbiota and can provide strategies for management of the atherosclerosis (53, 54). The consumption of red meat leads to the formation of TMAO by gut microbial activities. Thus, avoiding red meat is suggested for managing atherosclerosis (55, 56). In the case of chronic kidney disease (CKD), the prevalence of atherosclerotic events is higher. Higher TMAO levels are positively correlated with serum creatine and urea in CKD patients (57). Further, it is found that glucose intolerance susceptibility has an association with gut microbiota and non-caloric artificial sweeteners (NAS) can increase glucose intolerance (58). These artificial sweeteners are not absorbed by the gut and are thus speculated to interact directly with the gut microbiota and may affect physiological functions (59, 60). Thus, avoiding artificial sweeteners is considered a subset of personalized nutrition in such patients.

The gut microbes help in the catabolism of food, appetite alteration, and harvesting of nutrients. The live microbes in the form of “probiotics” when administered are useful in providing health benefits to the host. The microbes like *Bifidobacterium*, *E.coli*, and *Lactobacillus* are being used for disease management including gut dysbiosis, diarrhea, inflammatory bowel disease, Crohn’s disease, and recurrent *Clostridium difficile* infection (61, 62). The advancements in technology helped to develop strategies for microbiota transplantation, which has been proven to be useful in treating the illness associated with gut dysbiosis. Here, gut microbiota can be altered by a novel approach called fecal microbiota transplantation (FMT), a promising approach to alter gut microbiomes.

## 4 Precision nutrition and their characteristics

Each individual requires some specific amount of nutrients depending on genetic makeup, metabolisms, and microbiomes (63). Therefore, precision nutrition can be recommended for the individuals irrespective of the population level. Precision nutrition provides a detailed overview of the genetics, metagenome, microbiome, metabolome, and epigenetics of the individuals through latest next-generation analytical techniques. The generation of the analytical data and their assessment via computational-aided methods help in sharing the connection between the diet, lifestyle of the person, and health of the individuals (64).

Different populations of the world have some specific characteristics depending on cultivated crops, climate, and geographical location. These differences also lead to variations in the genetic makeup of the population. Although various governmental institutions have proposed different types of nutritional guidelines for the population, these guidelines give a brief overview of nutrient composition, nutrients needed, and a particular guideline for the population (65). Moreover, the differences in the population, regional diversity, and the genetic makeup of the person allow variation in the guidelines among different regions (66).

Recognizing the needs of individuals in view of precision nutrition is much challenging. The identification and development of nutritional advice for individuals require a broad analytical instrumentation, a computer-generated database, and extensive research analysis. Noteworthy, the differences in developmental stages, physiology, and health conditions contribute to the varied nutritional and caloric requirements of individuals. During the critical growth phase, adolescents require higher levels of proteins, and carbohydrates in comparison to adults (67). Similarly, pregnant women require some unique diet plan to nourish the growing infant. However, the elderly person facing challenges related to bone and muscle aging is advised to consume foods abundant in bioavailable calcium and proteins to address these age-related issues (68). Additionally, with the passage of age, physical activity often decreases, making it necessary to opt the foods having lower calorie density to prevent weight gain and obesity. On the contrary, individuals in demanding professions like army men, fire fighters, and athletes require calorie-dense, nutrient-rich foods (69). Variations in the gastrointestinal physiological patterns of the individual persons may also necessitate different nutrient profiles and levels of digestibility in their food (70).

The symbiotic relationship between the human body and the intestinal flora is crucial for the maintenance of normal physiology and metabolic function. The variations in normal microflora including intestinal flora of the individuals can led to genetic differences (71). However, the some of the studies have revealed that the composition and diversity of the intestinal microflora is influenced by the diets standing out as one of the most easily modifiable external factors (72).

The variations in dietary patterns significantly influence the microflora composition of population (73). For example, Japanese individuals harbor a distinct microbial strain in their intestinal tracts capable of secreting metabolic enzymes capable to digest algal constituents, a trait attributed to regular consumption of seaweed (74). This study underscores the direct impact of dietary interventions on the intestinal microflora and their functional aspect required for the normal functioning of human health.

## 5 Nutritional omics and systems biology

In the early stages of food nutrition, nutrigenomics research primarily focused on the exploration of new mechanisms related to nutrition and diet through the application of transcriptomics. The oxidative stress and inflammation can lead to changes in the transcriptomeas compared to the normal. So, the investigation of the proteome and the transcriptome will help in understanding the food patterns of a healthy person (75). Similarly, the advanced metabolomic approaches are gaining prominence in nutrition research because metabolites directly reflect the products of dietary intake and metabolism, which further help in the assessment of the relation and molecular pathways between diet metabolisms and the diseases (17).

The metabolism of the food in the human body is a complex phenomenon. Different metabolomic approaches may lead to identification of the new metabolites in the body that have prominent role in the functioning of colonic microflora and therefore impact on normal functioning of the human health (76). For example, in his study Rådjursöga et al. (77) reported that intake of breakfast cereals leads to enhancement of some specific amino acids like proline, tyrosine, N-acetylaminoacids in serum of the adults human body. However, the ingestion of ham and eggs leads to the enhancement of creatine, methanol, and isoleucine in the serum. Similarly, the lipid peroxidation metabolites considered as the markers to differentiate stable angina pectoris and myocardial infarction (78).

The metabolomics study has also provided an insight into individual’s biological age (79). Under the conditions of chronic diseases, the biological age becomes older than the actual age. The dietary intervention indicated that the ingested food and metabolites thereof affect the metagenome, metabolome, and transcriptome. Thus, the detection of changes in the metabolome, transcriptome or the metagenome at the early stages of the disease development can be helpful in developing the formulation of the new dietary patterns as a part of precision nutrition to achieve the goals of human health. Nutriomics involves the investigation of interactions between the human diet and genes, and their influence on the human health at the both molecular and population levels. This knowledge serves as a basis for creating personalized dietary interventions and healthcare strategies by analyzing individual’s genome structures. In the early stages, nutriomics research primarily focused on exploration of new mechanisms concerned with the nutrition through the application of transcriptomics and proteomics (17).

Modern food industry is facing a significant challenge in terms of food safety and waste, given the global production and transportation of ingredients and food items (80). It is imperative for the nutraceutical and food industries as well as government to adopt a plan (i) to prevent the food contamination, (ii) control minimize postharvest storage loss from the pathogenic microbes, and (iii) minimize the application of hazardous chemicals. In addition, authorities should introduce plans to enhance the nutrients composition of the food in order to maintain health and improve immunity.

Nowadays, the food industries and the academia are searching for alternative of various food ingredients from the proteins rich animal products like red meat, fish, eggs to plant based products, fermentation products, and cell cultures (65). Such kind of shift in food pattern may help in reducing biodiversity loss, greenhouse gas emission, and the maintenance of natural resources (81). However, it is crucial that foods derived from alternative protein sources are not only environmentally friendly but also nutritionally sound. While the macronutrient composition of many alternative protein sources resembles that of animal-derived foods, considerations, along with digestibility, are important. Additionally, the affordability, convenience, and taste of the food products constituted of alternative protein source are crucial factors of consumer acceptance (65).

## 6 The latest strategy to reduce the diet burden

The quality of the diet has major influence on the health of human beings. The population served with improved diet quality showed better resistance against the diseases and infections with improved health outcomes (82, 83). However, the healthy and the beneficial pattern of diets are the results of the presence of nutritional factors in the food. For instance, nowadays the healthy nutrient foods consisting of fresh fruits, vegetables, and fish are considered as the rich source of vitamins, minerals, antioxidants, etc. (83). However, the processed meat, refined grains, and sugar rich drinks have been considered as an unhygienic or unhealthy dietary pattern (84). The nutrients rich foods are considered to support the growth of gut microbiome and directly or indirectly affect the microbiome functioning with the resultant prevention of chronic diseases (85, 86).

Although the impact of nutritional content on health may be observed at any stage of disease, diet quality has been considered as one of the prime factors during diseases developments and the chances of infection can be reduced by modifying the diet pattern (87, 88). Further, the nutrient composition of the diet also impacts the recovery after the surgery (89). In addition, the diet quality also play pivotal role in aging (90). The evidence underscores the crucial role of diet quality, encompassing the composition of macro and micronutrients, in maintaining overall health. Diet influences disease occurrence, complication development, disease management, recovery, and the quality of life across a spectrum of health conditions (91–95). Dietary intervention trials frequently demonstrate that improving diet quality leads to enhanced health outcomes, independent of changes in weight (96). For instance, the plant based and low protein diet help control the chronic kidney diseases (97). The diet devoid of gluten and dairy products may have beneficial effects on children suffering from specific kidney disorders, and glomerulosclerosis (98). Similarly, the consumption of fish based diet enriched in omega-3 fatty acids could have positive effects on the management of IgA nephropathy (99). Thus, the inclusion of specific diets and components thereof as the part of precision nutrition may not only improve the human health, but also help reduce the globally rising risks of fatal diseases. The future of precision nutrition in the management of human diseases is presented in Table 1.

**Table 1:** Role of precision nutrition (PN) in the management of human disease.

Approaches of nutrition method	Omics and other technologies described	Disease managed	References
Precision nutrition	Nutrigenetics, metabolomics, metagenomics, behavioral aspects	Obesity and type 2 diabetes	Antwi (100)
Precision nutrition	Epigenetics, metabolomics, phenotypic anthropometrics	Aging and brain health	Chen and Small (101)
Precision nutrition	Environment, gut microflora, epigenetics	Female reproductive health	Dumesic et al. (102)
Precision nutrition	Lipoprotein analysis	Cardiovascular diseases	Hong et al. (103)
Precision nutrition	Genomics, proteomics	SARS-CoV-2	Naidu et al. (104)
Precision nutrition	The Genome, epigenome, and microbiome	Treatment of cardiovascular diseases	Ordovás (105)
Precision nutrition and omics tools approach	Genomics, epigenomics and metabolomics	Lactose intolerance management	Pratelli et al. (106)
Precision nutrition	Nutrigenomic, and Nutriepigenetic	Chronic diseases linked to obesity	Ramos-Lopez et al. (107)
Precision nutrition and dietary intervention	Metabolic phenotyping	Cardio-metabolic health management	Trouwborst et al. (108)
Precision nutrition	Biomarkers of food consumed, microbiome, lipidomics, genomics, transcriptomics	Obesity	Ulusoy-Gezer and Rakıcıoğlu (109)
Precision nutrition	Genomics, metabolomics, and gut microbiome technologies	Type 2 diabetes	Wang and Hu (110)
Precision nutrition	Target delivery of food functional factors	Chronic diseases like cancer, obesity, atherosclerosis	Yu et al. (16)

In the last few years, various international organization like Food and Drug Administration (FDA), started to issue a national guidelines to obtain the healthier food and nutrition information for their population. The enhanced information concerned with the healthier foods and nutrition profile will help in improving the health and wellness.^1^

Recently USA government released a national strategy to identify the nutrient profile of the food and improve the health hygiene so as to improve everyone’s health and wellness up to 2030 and control the diet related diseases, especially the obesity, diabetes, and hypertension.^2^ In light of the meetings held, the government encouraged the population to reduce the intake of sodium and sugar in the food, because the level of salt and sugar play a prominent role in diseases development. it has been observed that most of the population of U.S do not use enough amounts of healthy foods like fruits, dairy products, whole grains and also consume higher amount of sodium and sugar in the food. In the last few years, the world has faced the challenges of pandemic and huge number of global population. The developed countries were also hot spot of that pandemic. The poor nutrition and weak immune system are one of the major causes of such infections. Beside these, other diet specific epidemic like obesity, diabetes, and other cardiovascular diseases are also recognized to affect the U.S. population.

## 7 Machine learning and other digital technologies based approaches

Recent advances in digital technologies like machine learning (ML), deep learning, big data and artificial intelligence (AI) play a pivotal role in progression and usage of precision nutrition. Machine learning (ML) with the help of current digital technologies can venture into the unknown of nutraceuticals, identify valuable nutrients within our food and personalize the nutritional requirements. Machine learning can be quite useful in gathering dietary data inputs such as metabolomics, meal timing, meal uptake, physical activities, nutrigenomics, health conditions, genetics and personal health data. Further integrating the dietary data inputs, it can make a maximum likelihood prediction about the outcome using ML algorithms. The four types of ML considered in precision nutrition are supervised learning, unsupervised learning, semi-supervised learning and reinforcement learning (111). ML applies multiple algorithms, linear and logistic regression, data clustering analysis, artificial neural networks, deep learning, principal component analysis and data assessment to build a computational model for maximum output in understanding, characterization and precision nutrition in chronic dietary diseases (27, 112). Indeed, it has emerged as a quick solution to gather useful information from omics data, interpret it and predict the personalized dietary requirement and management. Moreover, it can also act as a biomarker monitoring tool assessing dietary intake, correlating with current health conditions and recommending precise nutraceutical for each individuals (27).

One of the recent example of application of ML in precision nutrition is the PREDICT 1 clinical trial (PersonalisedREsponses to Dietary Composition Trial; NCT03479866), where an ML model “robust to overfitting” was developed that predicted both triglyceride and glycemic responses to food intake. The predictions can further be used for precision nutrition and preparing dietary plans (113). Similarly, in a personalized diet study, hypocaloric diet was provided to overweight patients with pre-diabetic and moderately controlled diabetes type-2 using an ML based algorithm (114). In a comparison between statistical and machine-learning techniques used to evaluate the cardio-metabolic risks observed with dietary intake for 10 years (ATTICA study), ML techniques (k-nearest-neighbor’s algorithm and random-forests decision tree) were found to be superior than traditional linear regression model (115). ML algorithms have also been found useful in predicting the hereditary and environmental risks of lifestyle diseases, gene–gene, gene-nutrition interactions, change in microbiota and factors affecting the nutraceuticals (27, 116).

Artificial intelligence has unleashed a new arena in every possible field, including precision nutrition. AI-driven food interventions can diagnose the disease, assess the morbidity and mortality risk of a patient, discuss disease outbreak and surveillance and help in policy and planning using precision nutrition (117). AI, along with ML, can revolutionize the field of nutritional epidemiology in terms of precise nutrition measurement and addition of complex tools to model complexity of diet (118). Recently, Lee et al. (116) reported about two AI models (semantic and nutritional analysis models) which were developed and integrated into precision nutrition analysis using five different algorithms. The results showed that AI models could be used for nutritional survey and precision nutrition with high accuracy and reliability (116). Apart from working efficiently for large datasets, AI combined with big data and ML can generate nutrition-driven hypothesis and test them accurately, accounting for multiple factors at the same time (119).

Advancement in analytical and digital techniques have also helped to monitor and interpret nutraceutical data simultaneously. A number of smart phone- based apps have been developed which are replacing the traditional pen and paper-based databases for precision nutrition (120). Non-invasive wearable chemical and physical sensors and mobile and app-based electrochemical sensors can provide accurate real-time monitoring of dietary behavior changes toward a managed nutritional balance (121). Similarly, analytical techniques such as sensor-based vibrational spectroscopy, Raman, and terahertz spectroscopy, as well as hyperspectral imaging coupled with data analysis (ML, AI, and big data), can not only generate huge set of nutritional data on fluid-based biomarkers but also cut down the incurred cost substantially (19). Indeed, the dual use of continuous health monitoring and evaluation of continuously generated dynamic data can identify and provide recommendations on dietary requirements, which set a bright future for precision nutrition.

Despite being able to bring about revolutionary changes in precision nutrition by MI, AI and digital technologies, they still need certain hurdles huddles to overcome. Guidelines, and recommendations on the uses of AI are not very specific yet, and it can be misused easily. The generation of large set of data will further require protection of intellectual property and personal information. Further, ethical issue related to the use of AI also needs to be addressed. Collaboration between academics, industry and government and continuous upgrading of guidelines will further be required to AI-assisted precision nutrition.

## 8 Conclusion

Precision nutrition has been considered as one of the emerging approach in the field of human health management. The goal of precision nutrition research is to provide nutritional advice to individuals or populations. Although the nutritional profile that needs to be explored varies and depends upon genetics, sex, age, and geographical locations, it is now considered superior to generic advice. Currently, treatment of various human ailments is based on different therapeutic approaches, including traditional and modern medicine system. The continuously and gradually evolving disciplines of genomics in relation to nutrition have elucidated the importance of genetic variations, epigenetic information, and expression of myriads of genes in disease progression, apart from their involvement in modulating therapeutic responses. Although precision nutrition now includes the integrative study of latest omics of Nutrigenomics *like* metagenomics, metabolomics, and next-generation sequencing to understand the relationship between nutrition profile and human health management. However, the management of huge amounts of data generated by omics approaches is still a challenging task and warrants the application of latest artificial intelligence and machine learning tools for in depth understanding.

## Author contributions

VS: Conceptualization, Methodology, Writing – original draft. X-HH: Funding acquisition, Writing – review & editing. AS: Data curation, Writing – review & editing. MS: Data curation, Formal analysis, Visualization, Writing – review & editing. PV: Writing – review & editing. RS: Data curation, Writing – review & editing. NJ: Writing – review & editing. MK: Writing – review & editing. SS: Writing – review & editing. ZW: Funding acquisition, Writing – review & editing. AK: Conceptualization, Methodology, Writing – original draft, Writing – review & editing.

## Footnotes

^1^ https://www.fda.gov/food/food-labeling-nutrition/fdas-nutrition-initiatives

^2^ https://health.gov/our-work/nutrition-physical-activity/white-house-conference-hunger-nutrition-and-health

## References

[1] Mascie-Taylor CN Karim E. The burden of chronic disease. Science. (2003) 302:1921–2. doi: 10.1126/science.1092488, PMID: 14671291

[2] Nugent R. Chronic diseases in developing countries. Ann N Y Acad Sci. (2008) 1136:70–9. doi: 10.1196/annals.1425.027 18579877

[3] Lopez AD Mathers CD Ezzati M Jamison DT Murray CJ. Global and regional burden of disease and risk factors, 2001: systematic analysis of population health data. Lancet. (2006) 367:1747–57. doi: 10.1016/S0140-6736(06)68770-9, PMID: 16731270

[4] LeVatte M Keshteli AH Zarei P Wishart DS. Applications of metabolomics to precision nutrition. Lifestyle Genom. (2022) 15:1–9. doi: 10.1159/000518489, PMID: 34518463

[5] Blasbalg TL Hibbeln JR Ramsden CE Majchrzak SF Rawlings RR. Changes in consumption of omega-3 and omega-6 fatty acids in the United States during the 20th century. Am J Clin Nutr. (2011) 93:950–62. doi: 10.3945/ajcn.110.006643 21367944 PMC3076650

[6] Chilton MM Rabinowich JR Woolf NH. Very low food security in the USA is linked to exposure to violence. Public Health Nutr. (2014) 17:73–82. doi: 10.1017/S1368980013000281, PMID: 23432921 PMC10282483

[7] Popkin BM. Global nutrition dynamics: the world is shifting rapidly toward a diet linked with noncommunicable diseases. Am J Clin Nutr. (2006) 84:289–98. doi: 10.1093/ajcn/84.2.289 16895874

[8] Rayate AS Nagoba BS Mumbre SS Mavani HB Gavkare AM Deshpande AS. Current scenario of traditional medicines in the management of diabetic foot ulcers: a review. World J Diabetes. (2023) 14:1–16. doi: 10.4239/wjd.v14.i1.1 36684382 PMC9850800

[9] Birhan YS. Traditional zootherapeutic prescriptions employed in the management of neurological and related disorders in Ethiopia. Acta Ecol Sin. (2023) 43:585–95. doi: 10.1016/j.chnaes.2022.09.007

[10] Bairagi B Raghuwanshi RS. Immune-boosting properties of Ayurvedic formulations In: Immune-boosting nutraceuticals for better human health. New York: Apple Academic Press (2024). 333–54.

[11] Tsai K Ma H Liang TZ Xing Y Chung S Dorff T. The combined effect of immune checkpoint inhibitors and tyrosine kinase inhibitors on thyroid function. Thyroid. (2024) 34:158–66. doi: 10.1089/thy.2023.0542, PMID: 38069567 PMC10884548

[12] Che CT George V Ijinu TP Pushpangadan P Andrae-Marobela K. Traditional medicine In: Pharmacognosy. Massachusetts, USA: Academic Press (2024). 11–28.

[13] Zampieri F. The impact of modern medicine on human evolution In: On human nature. Cambridge, Massachusetts, USA: Academic Press (2017). 707–27.

[14] Koul A Gangar SC Sandhir V. Pitfalls in the journey from traditional to modern medicine. Nat. Prod. Radiance. (2005) 4:6–13.

[15] Voruganti VS. Precision nutrition: recent advances in obesity. Physiology. (2023) 38:42–50. doi: 10.1152/physiol.00014.2022, PMID: 36125787 PMC9705019

[16] Yu X Abd El-Aty AM Su W Tan M. Advancements in precision nutrition: steady-state targeted delivery of food functional factors for nutrition intervention in chronic diseases. Food Saf Health. (2023) 1:22–40. doi: 10.1002/fsh3.12006

[17] Brennan L de Roos B. Role of metabolomics in the delivery of precision nutrition. Redox Biol. (2023) 65:102808. doi: 10.1016/j.redox.2023.102808, PMID: 37423161 PMC10461186

[18] Tebani A Bekri S. Paving the way to precision nutrition through metabolomics. Front Nutr. (2019) 6:41. doi: 10.3389/fnut.2019.00041 31024923 PMC6465639

[19] Dongdong N Cozzolino D. Possibilities on the application of vibrational spectroscopy and data analytics in precision nutrition. TrAC Trends Anal Chem. (2023) 163:117067. doi: 10.1016/j.trac.2023.117067

[20] Adams SH Li Z Heber D. The role of metabolomics profiles in precision nutrition In: Precision nutrition. Massachusetts, USA: Academic Press (2024). 77–90.

[21] Bordoni L Gabbianelli R. Primers on nutrigenetics and nutri (epi) genomics: origins and development of precision nutrition. Biochimie. (2019) 160:156–71. doi: 10.1016/j.biochi.2019.03.006, PMID: 30878492

[22] Li X Qi L. Epigenetics in precision nutrition. J Pers Med. (2022) 12:533. doi: 10.3390/jpm12040533, PMID: 35455649 PMC9027461

[23] Sawicki C Haslam D Bhupathiraju S. Utilizing the precision nutrition toolkit in the path towards precision medicine. Proc Nutr Soc. (2023) 82:359–69. doi: 10.1017/S0029665123003038, PMID: 37475596

[24] Chaikijurajai T Tang WW. Gut microbiome and precision nutrition in heart failure: hype or hope? Curr Heart Fail Rep. (2021) 18:23–32. doi: 10.1007/s11897-021-00503-4, PMID: 33559845

[25] Martínez-Garay C Djouder N. Dietary interventions and precision nutrition in cancer therapy. Trends Mol Med. (2023) 29:489–511. doi: 10.1016/j.molmed.2023.04.004, PMID: 37263858

[26] Corzo L Fernández-Novoa L Carrera I Martínez O Rodríguez S Alejo R. Nutrition, health, and disease: role of selected marine and vegetal nutraceuticals. Nutrients. (2020) 12:747. doi: 10.3390/nu12030747, PMID: 32168971 PMC7146393

[27] Livingstone KM Ramos-Lopez O Perusse L Kato H Ordovas JM Martínez JA. Precision nutrition: a review of current approaches and future endeavors. Trends Food Sci Technol. (2022) 128:253–64. doi: 10.1016/j.tifs.2022.08.017

[28] Sebek M Menichetti G. Network science and machine learning for precision nutrition In: Precision nutrition. Massachusetts, USA: Academic Press (2024). 367–402.

[29] Mickelson B Herfel TM Booth J Wilson RP. Nutrition In: The laboratory rat. 3rd ed. Massachusetts, USA: Academic Press (2019). 243–347.

[30] Clarke ED Rollo ME Pezdirc K Collins CE Haslam RL. Urinary biomarkers of dietary intake: a review. Nutr Rev. (2020) 78:364–81. doi: 10.1093/nutrit/nuz048, PMID: 31670796

[31] Li J Guasch-Ferré M Chung W Ruiz-Canela M Toledo E Corella D. The Mediterranean diet, plasma metabolome, and cardiovascular disease risk. Eur Heart J. (2020) 41:2645–56. doi: 10.1093/eurheartj/ehaa209, PMID: 32406924 PMC7377580

[32] Kim H Rebholz CM. Metabolomic biomarkers of healthy dietary patterns and cardiovascular outcomes. Curr Atheroscler Rep. (2021) 23:1–12. doi: 10.1007/s11883-021-00921-8 33782776

[33] Palmnäs M Brunius C Shi L Rostgaard-Hansen A Torres NE González-Domínguez R. Perspective: metabotyping—a potential personalized nutrition strategy for precision prevention of cardiometabolic disease. Adv Nutr. (2020) 11:524–32. doi: 10.1093/advances/nmz121, PMID: 31782487 PMC7231594

[34] Hillesheim E Ryan MF Gibney E Roche HM Brennan L. Optimization of a metabotype approach to deliver targeted dietary advice. Nutr Metab. (2020) 17:1–12. doi: 10.1186/s12986-020-00499-z PMC7523294 33005208

[35] Mills S Stanton C Lane JA Smith GJ Ross RP. Precision nutrition and the microbiome, part I: current state of the science. Nutrients. (2019) 11:923. doi: 10.3390/nu11040923, PMID: 31022973 PMC6520976

[36] Arumugam N Kalavathi P Mahalingam PU. Lignin database for diversity of lignin-degrading microbial enzymes (LD2L). Res Biotechnol. (2014) 5:13–8.

[37] Ferguson LR. Nutrigenomics approaches to functional foods. J Am Diet Assoc. (2009) 109:452–8. doi: 10.1016/j.jada.2008.11.024 19248861

[38] Farhud D Shalileh M. Phenylketonuria and its dietary therapy in children. Iran J Pediatr. (2008) 18:88–98.

[39] Trujillo E Davis C Milner J. Nutrigenomics, proteomics, metabolomics, and the practice of dietetics. J Am Diet Assoc. (2006) 106:403–13. doi: 10.1016/j.jada.2005.12.002, PMID: 16503231

[40] Goh KI Cusick ME Valle D Childs B Vidal M Barabási AL. The human disease network. Proc Natl Acad Sci USA. (2007) 104:8685–90. doi: 10.1073/pnas.0701361104, PMID: 17502601 PMC1885563

[41] Farhud DD Yeganeh MZ. Nutrigenomics and nutrigenetics. Iran J Public Health. (2010) 39:1–14. PMID: 23113033 PMC3481686

[42] Costello KR Schones DE. Chromatin modifications in metabolic disease: potential mediators of long-term disease risk. Wiley Interdiscip Rev Syst Biol Med. (2018) 10:1416. doi: 10.1002/wsbm.1416 PMC6002879 29369528

[43] Dandare A Khan MJ Naeem A Liaquat A. Clinical relevance of circulating non-coding RNAs in metabolic diseases: emphasis on obesity, diabetes, cardiovascular diseases and metabolic syndrome. Genes Dis. (2023) 10:2393–413. doi: 10.1016/j.gendis.2022.05.022, PMID: 37554181 PMC10404886

[44] Ordovas JM Ferguson LR Tai ES Mathers JC. Personalised nutrition and health. BMJ. (2018):bmj.k2173. doi: 10.1136/bmj.k2173 29898881 PMC6081996

[45] Aggarwal N Kitano S Puah GR Kittelmann S Hwang IY Chang MW. Microbiome and human health: current understanding, engineering, and enabling technologies. Chem Rev. (2023) 123:31–72. doi: 10.1021/acs.chemrev.2c00431 36317983 PMC9837825

[46] Jameson JL Longo DL. Precision medicine—personalized, problematic, and promising. Obstetr Gynecol Surv. (2015) 70:612–4. 10.1056/NEJMsb1503104 26014593

[47] Hamburg MA Collins FS. The path to personalized medicine. N Engl J Med. (2010) 363:301–4. doi: 10.1056/NEJMp1006304, PMID: 20551152

[48] Moschonis G Michalopoulou M Tsoutsoulopoulou K Vlachopapadopoulou E Michalacos S Charmandari E. Assessment of the effectiveness of a computerised decision-support tool for health professionals for the prevention and treatment of childhood obesity. Results from a randomised controlled trial. Nutrients. (2019) 11:706. doi: 10.3390/nu11030706, PMID: 30917561 PMC6471646

[49] Petrie JR Guzik TJ Touyz RM. Diabetes, hypertension, and cardiovascular disease: clinical insights and vascular mechanisms. Can J Cardiol. (2018) 34:575–84. doi: 10.1016/j.cjca.2017.12.005, PMID: 29459239 PMC5953551

[50] Eggersdorfer M Kraemer K Cordaro JB Fanzo J Gibney M Kennedy E. Good nutrition: perspectives for the 21st century. Basel, Switzerland: Karger (2016).

[51] Zeevi D Korem T Zmora N Israeli D Rothschild D Weinberger A. Personalized nutrition by prediction of glycemic responses. Cell. (2015) 163:1079–94. doi: 10.1016/j.cell.2015.11.001, PMID: 26590418

[52] Benjamin EJ Blaha MJ Chiuve SE Cushman M Das SR Deo R. Heart disease and stroke statistics—2017 update: a report from the American Heart Association. Circulation. (2017) 135:e146–603. doi: 10.1161/CIR.0000000000000485, PMID: 28122885 PMC5408160

[53] Bennett BJ de AguiarVallim TQ Wang Z Shih DM Meng Y Gregory J. Trimethylamine-N-oxide, a metabolite associated with atherosclerosis, exhibits complex genetic and dietary regulation. Cell Metab. (2013) 17:49–60. doi: 10.1016/j.cmet.2012.12.011, PMID: 23312283 PMC3771112

[54] Ufnal M Zadlo A Ostaszewski R. TMAO: a small molecule of great expectations. Nutrition. (2015) 31:1317–23. doi: 10.1016/j.nut.2015.05.006, PMID: 26283574

[55] Rohrmann S Overvad K Bueno-de-Mesquita HB Jakobsen MU Egeberg R Tjønneland A. Meat consumption and mortality—results from the European prospective investigation into Cancer and nutrition. BMC Med. (2013) 11:1–12. doi: 10.1186/1741-7015-11-63 23497300 PMC3599112

[56] Zhu Y Li Q Jiang H. Gut microbiota in atherosclerosis: focus on trimethylamine N-oxide. APMIS. (2020) 128:353–66. doi: 10.1111/apm.13038, PMID: 32108960 PMC7318354

[57] Lombardo M Aulisa G Marcon D Rizzo G Tarsisano MG Di Renzo L. Association of urinary and plasma levels of trimethylamine n-oxide (Tmao) with foods. Nutrients. (2021) 13:1426. doi: 10.3390/nu13051426, PMID: 33922680 PMC8145508

[58] Suez J Korem T Zeevi D Zilberman-Schapira G Thaiss CA Maza O. Artificial sweeteners induce glucose intolerance by altering the gut microbiota. Nature. (2014) 514:181–6. doi: 10.1038/nature13793, PMID: 25231862

[59] Ma J Chang J Checklin HL Young RL Jones KL Horowitz M. Effect of the artificial sweetener, sucralose, on small intestinal glucose absorption in healthy human subjects. Br J Nutr. (2010) 104:803–6. doi: 10.1017/S0007114510001327, PMID: 20420761

[60] Hasan HM Alkass SY de Oliveira DSP. Impact of long-term cyclamate and saccharin consumption on biochemical parameters in healthy individuals and type 2 diabetes mellitus patients. Medicina. (2023) 59:698. doi: 10.3390/medicina59040698, PMID: 37109657 PMC10146554

[61] Cuello-Garcia CA Brożek JL Fiocchi A Pawankar R Yepes-Nuñez JJ Terracciano L. Probiotics for the prevention of allergy: a systematic review and meta-analysis of randomized controlled trials. J Allergy Clin Immunol. (2015) 136:952–61. doi: 10.1016/j.jaci.2015.04.031, PMID: 26044853

[62] Coqueiro AY Raizel R Bonvini A Tirapegui J Rogero MM. Probiotics for inflammatory bowel diseases: a promising adjuvant treatment. Int J Food Sci Nutr. (2019) 70:20–9. doi: 10.1080/09637486.2018.1477123 29804478

[63] McClements DJ. Future foods: How modern science is transforming the way we eat (pp. 323–361). Basel: Springer. (2019).

[64] Liu F Li M Wang Q Yan J Han S Ma C. Future foods: alternative proteins, food architecture, sustainable packaging, and precision nutrition. Crit Rev Food SciNutr. (2023) 63:6423–44. doi: 10.1080/10408398.2022.2033683, PMID: 35213241

[65] McClements DJ. Future foods: a manifesto for research priorities in the structural design of foods. Food Function. (2020) 11:1933–45. doi: 10.1039/c9fo02076d 32141468

[66] Hernandez LM Blazer DG. The impact of social and cultural environments on health In: Genes, behavior, and the social environment: Moving beyond the nature/nurture debate. Washington, DC, USA: National Academies Press (US) (2006) 20669442

[67] Soliman A Alaaraj N Hamed N Alyafei F Ahmed S Shaat M. Nutritional interventions during adolescence and their possible effects. Acta Biomed. (2022) 93:e2022087. doi: 10.23750/abm.v93i1.12789 35315384 PMC8972883

[68] Jouanne M Oddoux S Noël A Voisin-Chiret AS. Nutrient requirements during pregnancy and lactation. Nutrients. (2021) 13:692. doi: 10.3390/nu13020692, PMID: 33670026 PMC7926714

[69] Pasiakos SM. Nutritional requirements for sustaining health and performance during exposure to extreme environments. Ann Rev Nutr. (2020) 40:221–45. doi: 10.1146/annurev-nutr-011720-122637, PMID: 32530730

[70] Sender R Fuchs S Milo R. Are we really vastly outnumbered? Revisiting the ratio of bacterial to host cells in humans. Cell. (2016) 164:337–40. doi: 10.1016/j.cell.2016.01.013, PMID: 26824647

[71] Vandeputte D Joossens M. Effects of low and high FODMAP diets on human gastrointestinal microbiota composition in adults with intestinal diseases: a systematic review. Microorganisms. (2020) 8:1638. doi: 10.3390/microorganisms8111638, PMID: 33114017 PMC7690730

[72] Sonnenburg ED Smits SA Tikhonov M Higginbottom SK Wingreen NS Sonnenburg JL. Diet-induced extinctions in the gut microbiota compound over generations. Nature. (2016) 529:212–5. doi: 10.1038/nature16504, PMID: 26762459 PMC4850918

[73] Thomas F Hehemann JH Rebuffet E Czjzek M Michel G. Environmental and gut bacteroidetes: the food connection. Front Microbiol. (2011) 2:93. doi: 10.3389/fmicb.2011.00093, PMID: 21747801 PMC3129010

[74] Hehemann JH Correc G Barbeyron T Helbert W Czjzek M Michel G. Transfer of carbohydrate-active enzymes from marine bacteria to the Japanese gut microbiota. Nature. (2010) 464:908–12. doi: 10.1038/nature08937, PMID: 20376150

[75] Van Ommen B Stierum R. Nutrigenomics: exploiting systems biology in the nutrition and health arena. Curr Opin Biotechnol. (2002) 13:517–21. doi: 10.1016/S0958-1669(02)00349-X 12459347

[76] Farag MA Abdelwareth A Sallam IE El Shorbagi M Jehmlich N Fritz-Wallace K. Metabolomics reveals the impact of seven functional foods on metabolic pathways in a gut microbiota model. J Adv Res. (2020) 23:47–59. doi: 10.1016/j.jare.2020.01.001, PMID: 32071791 PMC7016031

[77] Rådjursöga M Lindqvist HM Pedersen A Karlsson GB Malmodin D Brunius C. The 1 H NMR serum metabolomics response to a two-meal challenge: a cross-over dietary intervention study in healthy human volunteers. Nutr J. (2019) 18:1–12. doi: 10.1186/s12937-019-0446-2 30961592 PMC6454665

[78] Zhong S Li L Shen X Li Q Xu W Wang X. An update on lipid oxidation and inflammation in cardiovascular diseases. Free Radic Biol Med. (2019) 144:266–78. doi: 10.1016/j.freeradbiomed.2019.03.036 30946962

[79] Ordovas JM Berciano S. Personalized nutrition and healthy aging. Nutr Rev. (2020) 78:58–65. doi: 10.1093/nutrit/nuaa102 33259626

[80] Meybeck A Gitz V. Sustainable diets within sustainable food systems. Proc Nutr Soc. (2017) 76:1–11. doi: 10.1017/S0029665116000653 28195528

[81] Parodi A Leip A De Boer IJM Slegers PM Ziegler F Temme EH. The potential of future foods for sustainable and healthy diets. Nat Sustain. (2018) 1:782–9. doi: 10.1038/s41893-018-0189-7, PMID: 38998582

[82] Lai JS Hiles S Bisquera A Hure AJ McEvoy M Attia J. A systematic review and meta-analysis of dietary patterns and depression in community-dwelling adults. Am J Clin Nutr. (2014) 99:181–97. doi: 10.3945/ajcn.113.069880 24196402

[83] Parletta N Milte CM Meyer BJ. Nutritional modulation of cognitive function and mental health. J Nutr Biochem. (2013) 2013:725–43. doi: 10.1016/j.jnutbio.2013.01.002 23517914

[84] Simopoulos AP. Evolutionary aspects of diet: the omega-6/omega-3 ratio and the brain. Mol Neurobiol. (2011) 44:203–15. doi: 10.1007/s12035-010-8162-0, PMID: 21279554

[85] Akbaraly TN Brunner EJ Ferrie JE Marmot MG Kivimaki M SinghManoux A. Dietary pattern and depressive symptoms in middle age. Br J Psychiatry. (2009) 195:408–13. doi: 10.1192/bjp.bp.108.058925, PMID: 19880930 PMC2801825

[86] Kouris-Blazos A Itsiopoulos C. Low all-cause mortality despite high cardiovascular risk in elderly Greek-born Australians: attenuating potential of diet? Asia Pac J Clin Nutr. (2014) 23:532–44. doi: 10.6133/apjcn.2014.23.4.16 25516310

[87] Opie RS O’Neil A Itsiopoulos C Jacka FN. The impact of whole-of-diet interventions on depression and anxiety: a systematic review of randomised controlled trials. Public Health Nutr. (2013) 18:2074–93. doi: 10.1017/S1368980014002614 PMC10271872 25465596

[88] Sánchez-Villegas A Martínez-González MA Estruch R Salas-SalvadóJ CD Covas MI Arós F. Mediterranean dietary pattern and depression: the PREDIMED randomized trial. BMC Med. (2013) 11:208. doi: 10.1186/1741-7015-11-208 24229349 PMC3848350

[89] Cerantola Y Grass F Cristaudi A Demartines N Schäfer M Hübner M. Perioperative nutrition in abdominal surgery: recommendations and reality. Gastroenterol Res Pract. (2011):739347. doi: 10.1155/2011/739347, PMID: 21687620 PMC3113259

[90] HodgeAMO'DeaKEnglishDRGilesGGFlickerL. Dietary patterns as predictors of successful ageing. J Nutr Health Aging. (2014) 8:221–7. doi: 10.1007/s12603-013-0405-024626747

[91] Sofi F Cesari F Abbate R Gensini GF Casini A. Adherence to Mediterranean diet and health status: meta-analysis. BMJ. (2008) 337:a1344. doi: 10.1136/bmj.a1344, PMID: 18786971 PMC2533524

[92] Estruch R Ros E Salas-Salvadó J Covas MI Corella D Arós F. Primary prevention of cardiovascular disease with a Mediterranean diet. N Engl J Med. (2013) 368:1279–90. doi: 10.1056/NEJMoa1200303, PMID: 29897867

[93] Esposito K Maiorino M Ceriello A Giugliano D. Prevention and control of type 2 diabetes by Mediterranean diet: a systematic review. Diab Res Clin Pract. (2010) 89:97–102. doi: 10.1016/j.diabres.2010.04.019 20546959

[94] SM Thorton E Smarkola C Kopacz SM Ishoof SB. Perinatal outcomes in nutritionally monitored obese pregnant women: RCT. J Natl Med Assoc. (2009) 101:569–77. doi: 10.1016/s0027-9684(15)30942-1 19585925

[95] Heizer WD Southern S McGovern S. The role of diet in symptoms of irritable bowel syndrome in adults: a narrative review. J Am Diet Assoc. (2009) 109:1204–14. doi: 10.1016/j.jada.2009.04.012 19559137

[96] Itsiopoulos C Brazionis L Kaimakamis M Cameron M Best JD O’Dea K. Can the Mediterranean diet lower HbA1c in type 2 diabetes? Results from a randomized cross-over study. Nutr Metab Cardiovasc Dis. (2011) 21:740–7. doi: 10.1016/j.numecd.2010.03.005 20674309

[97] Kalantar-Zadeh K Joshi S Schlueter R Cooke J Brown-Tortorici A Donnelly M. Plant-dominant low-protein diet for conservative management of chronic kidney disease. Nutrients. (2020) 12:1931. doi: 10.3390/nu12071931, PMID: 32610641 PMC7400005

[98] Leon J Pérez-Sáez MJ Uffing A Murakami N Watanabe A Cureton P. Effect of combined gluten-free, dairy-free diet in children with steroid-resistant nephrotic syndrome: an open pilot trial. Kidney Int Rep. (2018) 3:851–60. doi: 10.1016/j.ekir.2018.02.011, PMID: 30116795 PMC6093178

[99] Coppo R. The gut-renal connection in IgA nephropathy In: Seminars in nephrology, vol. 38. Massachusetts, USA: WB Saunders (2018). 504–12. 30177022 10.1016/j.semnephrol.2018.05.020

[100] Antwi J. Precision nutrition to improve risk factors of obesity and type 2 diabetes. Curr Nutr Rep. (2023) 12:679–94. doi: 10.1007/s13668-023-00491-y, PMID: 37610590 PMC10766837

[101] Chen ST Small GW. Precision nutrition in aging and brain health In: Precision nutrition. Massachusetts, USA: Academic Press (2024). 241–76.

[102] Dumesic D Chazenbalk G Heber D. Precision nutrition in female reproductive health In: Precision nutrition. Massachusetts, USA: Academic Press (2024). 227–40.

[103] Hong BV Agus JK Tang X Zheng JJ Romo EZ Lei S. Precision nutrition and cardiovascular disease risk reduction: the promise of high-density lipoproteins. Curr Atheroscler Rep. (2023) 25:663–77. doi: 10.1007/s11883-023-01148-5, PMID: 37702886 PMC10564829

[104] Naidu AS Wang CK Rao P Mancini F Clemens RA Wirakartakusumah A. Precision nutrition to reset virus-induced human metabolic reprogramming and dysregulation (HMRD) in long-COVID. npj Sci Food. (2024) 8:19. doi: 10.1038/s41538-024-00261-2, PMID: 38555403 PMC10981760

[105] Ordovás J. A multifaceted approach to precision nutrition: the genome, Epigenome, and microbiome in the prevention and therapy of cardiovascular diseases In: Precision nutrition. Massachusetts, USA: Academic Press (2024). 181–200.

[106] Pratelli G Tamburini B Badami GD Lo Pizzo M De Blasio A Carlisi D. Cow’s Milk: a benefit for human health? Omics tools and precision nutrition for lactose intolerance management. Nutrients. (2024) 16:320. doi: 10.3390/nu16020320, PMID: 38276558 PMC10819418

[107] Ramos-Lopez O Milagro FI Allayee H Chmurzynska A Choi MS Curi R. Guide for current nutrigenetic, nutrigenomic, and nutriepigenetic approaches for precision nutrition involving the prevention and management of chronic diseases associated with obesity. Lifestyle Genom. (2017) 10:43–62. doi: 10.1159/000477729, PMID: 28689206

[108] Trouwborst I Gijbels A Jardon KM Siebelink E Hul GB Wanders L. Cardiometabolic health improvements upon dietary intervention are driven by tissue-specific insulin resistance phenotype: a precision nutrition trial. Cell Metab. (2023) 35:71–83. doi: 10.1016/j.cmet.2022.12.002, PMID: 36599304

[109] Ulusoy-Gezer HG Rakıcıoğlu N. The future of obesity management through precision nutrition: putting the individual at the center. Curr Nutr Rep. (2024):1–23. doi: 10.1007/s13668-024-00550-y 38806863 PMC11327204

[110] Wang DD Hu FB. Precision nutrition for prevention and management of type 2 diabetes. Lancet Diab Endocrinol. (2018) 6:416–26. doi: 10.1016/S2213-8587(18)30037-8, PMID: 29433995

[111] Kirk D Catal C Tekinerdogan B. Precision nutrition: a systematic literature review. Comput Biol Med. (2021) 133:104365. doi: 10.1016/j.compbiomed.2021.104365 33866251

[112] KW DG Kuiper P DeSilvio T Pleuss JD Miller R Roginski JW. A review of machine learning in obesity. Obes Rev. (2018) 19:668–85. doi: 10.1111/obr.12667, PMID: 29426065 PMC8176949

[113] Berry SE Valdes AM Drew DA Asnicar F Mazidi M Wolf J. Human postprandial responses to food and potential for precision nutrition. Nat Med. (2020) 26:964–73. doi: 10.1038/s41591-020-0934-0 32528151 PMC8265154

[114] Popp C Hu L Wang C Curran M Li H Kharmats A. A randomized clinical trial to compare a precision nutrition intervention targeting a reduction in postprandial glycemic response to meals with a low-fat diet for weight loss. Curr Dev Nutr. (2022) 6:1122. doi: 10.1093/cdn/nzac078.016

[115] Panaretos D Koloverou E Dimopoulos AC Kouli GM Vamvakari M Tzavelas G. A comparison of statistical and machine-learning techniques in evaluating the association between dietary patterns and 10-year cardiometabolic risk (2002–2012): the ATTICA study. Br J Nutr. (2018) 120:326–34. doi: 10.1017/S0007114518001150, PMID: 29789037

[116] Lee HA Huang TT Yen LH Wu PH Chen KW Kung HH. Precision nutrient management using artificial intelligence based on a digital data collection framework. Appl Sci. (2022) 12:4167. doi: 10.3390/app12094167

[117] Schwalbe N Wahl B. Artificial intelligence and the future of global health. Lancet. (2020) 395:1579–86. doi: 10.1016/S0140-6736(20)30226-9 32416782 PMC7255280

[118] Morgenstern JD Rosella LC Costa AP de Souza RJ Anderson LN. Perspective: big data and machine learning could help advance nutritional epidemiology. Adv Nutr. (2021) 12:621–31. doi: 10.1093/advances/nmaa183, PMID: 33606879 PMC8166570

[119] Morton SU Leyshon BJ Tamilia E Vyas R Sisitsky M Ladha I. A role for data science in precision nutrition and early brain development. Front Psych. (2022) 13:892259. doi: 10.3389/fpsyt.2022.892259, PMID: 35815018 PMC9259898

[120] Mortazavi BJ Gutierrez-Osuna R. A review of digital innovations for diet monitoring and precision nutrition. J Diab Sci Technol. (2023) 17:217–23. doi: 10.1177/19322968211041356 PMC9846399 34467803

[121] Sempionatto JR Montiel VRV Vargas E Teymourian H Wang J. Wearable and mobile sensors for personalized nutrition. ACS Sens. (2021) 6:1745–60. doi: 10.1021/acssensors.1c00553, PMID: 34008960

